# Radiological identification of temporal lobe epilepsy using artificial intelligence: a feasibility study

**DOI:** 10.1093/braincomms/fcab284

**Published:** 2021-12-08

**Authors:** Ezequiel Gleichgerrcht, Brent Munsell, Simon S Keller, Daniel L Drane, Jens H Jensen, M Vittoria Spampinato, Nigel P Pedersen, Bernd Weber, Ruben Kuzniecky, Carrie McDonald, Leonardo Bonilha

**Affiliations:** 1 Department of Neurology, Medical University of South Carolina, Charleston, SC 29425, USA; 2 Department of Computer Science, University of North Carolina, Chapel Hill, NC 27599, USA; 3 Department of Psychiatry, University of North Carolina, Chapel Hill, NC 27599, USA; 4 Institute of Systems, Molecular and Integrative Biology, University of Liverpool, Liverpool L69 7BE, UK; 5 The Walton Centre NHS Foundation Trust, Liverpool L9 7LJ, UK; 6 Department of Neurology, Emory University, Atlanta, GA 30322, USA; 7 Center for Biomedical Imaging, Medical University of South Carolina, Charleston, SC 29425, USA; 8 Department of Radiology, Medical University of South Carolina, Charleston, SC 29425, USA; 9 Institute of Experimental Epileptology and Cognition Research, University of Bonn, Bonn 53113, Germany; 10 Department of Neurology, Hofstra University/Northwell, New York, NY 10075, USA; 11 Department of Psychiatry, University of California San Diego, La Jolla, CA 92093, USA

**Keywords:** artificial intelligence, temporal lobe epilepsy, convoluted neural network, structural neuroimaging

## Abstract

Temporal lobe epilepsy is associated with MRI findings reflecting underlying mesial temporal sclerosis. Identifying these MRI features is critical for the diagnosis and management of temporal lobe epilepsy. To date, this process relies on visual assessment by highly trained human experts (e.g. neuroradiologists, epileptologists). Artificial intelligence is increasingly recognized as a promising aid in the radiological evaluation of neurological diseases, yet its applications in temporal lobe epilepsy have been limited. Here, we applied a convolutional neural network to assess the classification accuracy of temporal lobe epilepsy based on structural MRI. We demonstrate that convoluted neural networks can achieve high accuracy in the identification of unilateral temporal lobe epilepsy cases even when the MRI had been originally interpreted as normal by experts. We show that accuracy can be potentiated by employing smoothed grey matter maps and a direct acyclic graphs approach. We further discuss the foundations for the development of computer-aided tools to assist with the diagnosis of epilepsy.

## Teaser

Gleichgerrcht et al. tested whether artificial intelligence can identify persons with temporal lobe epilepsy based on their brain MRI scans. They demonstrated that convoluted neural networks achieve high accuracy in the identification of these patients even when the MRI had been originally interpreted as normal by experts.

## Graphical Abstract

**Graphical Abstract:**
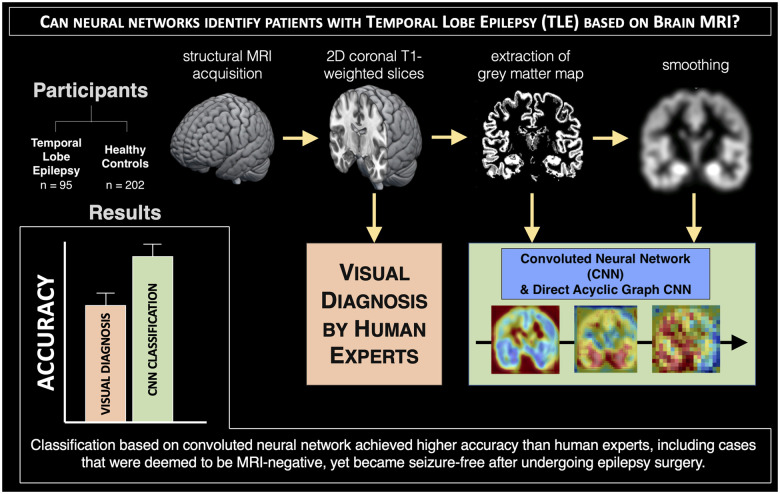

## Introduction

The diagnosis of epilepsy-related radiological abnormalities depends on the identification of subtle imaging features.^1^ Their accurate interpretation requires considerable expert training and can be prone to human error.^2^ Deep learning and convolutional neural networks (CNNs) have been increasingly recognized as promising aids in the radiological evaluation of neurological diseases such as Alzheimer’s disease, brain tumours, as well as other systemic conditions that rely on imaging or pathological findings, such as pneumonia or skin cancers.^3^ In spite of the high prevalence of epilepsy, artificial intelligence has not been equivalently explored for the radiological detection of epilepsy-related brain abnormalities.^4^

This gap is possibly related to several important characteristics that set epilepsy apart from other neurological conditions. First, even though epilepsy is prevalent, affecting approximately 1% of the world population, it is a heterogeneous disease. However, the most common form of epilepsy in adults is temporal lobe epilepsy (TLE). In addition to its high prevalence, TLE is also the most common form of drug-resistant epilepsy,^5^ incurring considerable healthcare costs. Identifying subtle radiographic findings associated with TLE can be critical to the neurological evaluation of epilepsies, especially for cases that may require surgical treatment. TLE is often part of the differential diagnosis. Given that imaging findings can be subtle in the various types of TLE, deep learning could aid in the analysis of imaging, having a wide-ranging impact in the diagnosis and treatment of epilepsy in general.

Second, the most frequent subtype of TLE, medial TLE (MTLE), is commonly associated with mesial temporal sclerosis (MTS), which is a histological abnormality, often with radiologic correlates, defined by cell loss and gliosis in the hippocampus. Radiographically, this cell loss is associated with reduced regional volume or loss of hippocampal internal structures, which can be appreciated on T_1_-weighted images,^6^ while gliosis is associated with increased T_2_ signal.^7^ Regional atrophy and increased T_2_ signal can be readily apparent in some instances, but many MTLE cases are not clearly recognized on visual inspection. Accordingly, manual or automated quantification of medial temporal atrophy through volume measurements relative to normative databases have been helpful in increasing diagnostic accuracy in some cases.^2^,^8^ Likewise, T_2_ signal quantification may also increase imaging-based diagnostic accuracy.^9^ Nonetheless, these approaches do not detect the pattern of tissue damage in MTLE, which has been shown to extend beyond both the hippocampus and the medial temporal region.^10^,^11^ These abnormalities could be important for the diagnosis of MTLE but are subtle and often not detectable by visual inspection of diagnostic images. Similarly, with the wide array of other TLEs, including basal, lateral neocortical and polar regional involvement, new approaches to image analysis are warranted. CNN offers a unique opportunity to detect relevant hippocampal and extra-hippocampal changes, which are otherwise imperceptible to the human eye, and leverage them for diagnosis.

Third, compared with other forms of neurological diseases with abnormal brain signal, such as brain tumors or demyelinating lesions, the abnormalities of TLE do not involve prominent distortion of brain anatomy or unambiguous lesions. Instead, they are composed by widely distributed changes that can often be quite subtle. Furthermore, normal individual differences in sulcal and gyral positioning or anatomy^12^ can pose an additional challenge in the correct identification of TLE-related abnormalities. In this context, approaches such as spatial normalization of statistical tissue maps can reduce individual variability and increase the sensitivity to consistent abnormalities.^13^

Fourth, because some MTLE-related abnormalities within the medial temporal region may be confined to small parcels of brain tissue, such as the hippocampal formation, entorhinal cortex, or perirhinal cortex,^14^ the conventional multilayered approach with progressively larger filters may ‘overlook’ important abnormalities. Therefore, CNN architectures that leverage smaller filter approaches and bypass the sequential filtering architecture, such as direct acyclic graphs (DAG), may be more sensitive to MTLE and TLE regional changes that do not lead to large scale distortions.^15^

Taking these four points in consideration, we aimed to assess the feasibility of artificial intelligence (specifically, using neural networks) in detecting unilateral TLE. This is a proof-of-concept evaluation aimed at testing the ability of CNNs to identify MTLE, using the gold-standard of patients with MTLE who underwent surgical treatment (medial temporal resection including anterior temporal lobectomy but also selective amygdalohippocampectomy or laser ablation) and became seizure free, thus providing undisputable confirmation of the MTLE as well as unilateral seizure onset. We focused on a large sample of well-defined such cases of unilateral (left sided only) MTLE to test the classification accuracy, sensitivity, and specificity of CNNs. Given the proof-of-concept approach, we focussed on left MTLE to avoid lateralization issues^16^ and to increase sample homogeneity in this initial study. Moreover, we evaluated the best CNN architecture to identify regional features that are well-known to be associated with TLE by testing conventional versus DAG architectures of CNN models. Finally, we tested whether regional feature importance of CNNs agreed with sites of MTLE-related pathology extensively described in the literature.

## Materials and Methods

### Participants

The study included a total of 95 patients with left-sided MTLE from three different sites, including the Medical University of South Carolina (MUSC, *n *=* *30), Emory University (*n *=* *33) and University of Bonn (*n *=* *32). Patient diagnosis was achieved following standard of care assessment batteries at each site, including neurophysiology and neuroimaging studies. Only patients for whom clinical semiology, radiographic findings, and neurophysiology were concordant and strongly suggestive of a left medial temporal focus were included. A total of 202 healthy controls (HC) were also recruited across all three sites (MUSC *n = *49, Emory *n *=* *74 and Bonn *n = *79) if they had no prior history of psychiatric or neurological disorders. The Institutional Review Board (IRB) approval for anonymized data collection and data sharing was obtained at each centre prior to enrollment into the consortia.

The TLE cohort included patients with visually detected hippocampal or medial temporal lobe atrophy (*n* = 48) and patients without visually identifiable abnormalities (*n* = 47). The patients with medial temporal lobe atrophy were evenly distributed across all sites. There were no patients with other abnormalities besides medial temporal lobe atrophy, such as neocortical focal cortical dysplasia, brain tumors, arachnoid cysts, or strokes. Among the patients with left TLE, the majority underwent resection surgery (*n *=* *59) varying from selective amygdalohippocampectomy to complete anterior temporal lobectomy or stereotactic laser ablation (*n *=* *36) for the treatment of epilepsy after the MRI used in this study was acquired. Among those, at least one year after surgery, 57 became seizure free postoperatively: *n* = 29 among patients with hippocampal atrophy and *n* = 28 among those without.

For clarity, we will hereafter refer to patients with visually identified hippocampal atrophy as ‘lesional’ TLE, and those without hippocampal atrophy as ‘non-lesional’.

### Diagnostic gold-standard

In this study, the gold standard for the diagnosis of left TLE were patients who became seizure-free at least one year after surgery (lesional or non-lesional). This is the most unequivocal diagnostic confirmation of the presence and lateralization of TLE. This is a crucial aspect of this study: the goal here is not to define a classifier that is as accurate as humans in identifying hippocampal atrophy. The translational benefit of such a tool would be limited. Instead, if a classifier can accurately identify patients who become seizure free with or without visually perceptible hippocampal atrophy, this is a confirmation of diagnostic accuracy using features beyond the hippocampus that are typically not appreciated on visual inspection and demonstrates its potential benefit as a decision support tool.

### MRI preprocessing and GM tissue segmentation

All images were acquired preoperatively on a 3 T MRI scanner. Scanner type and acquisition parameters varied across institutions, as follows:

- **MUSC**: Siemens Skyra 3T scanner, isotropic voxel size 1 mm, 12-channel head coil, TR = 2050–2250 ms, TE = 2.5–18 ms, FOV = 256–320 mm, flip angle 10°;
- **Emory**: Siemens Prisma 3T scanner, isotropic voxel size 0.8mm, 12-channel head coil, TR = 2300 ms, TE = 2.75 ms, TI = 1100ms, flip angle 8
- **Bonn**: Siemens Magnetom Trio 3T scanner, 8-channel head coil, isotropic voxel size of 1mm, TR = 650ms, TE = 3.97ms, TI = 650ms, flip angle 10°

Image preprocessing was performed to normalize all images in standard stereotaxic MNI space and to segment brain tissues. For normalization into standard space, we used the normalize function from the software package SPM with the following parameters: bias regularization = 0.0001, bias FWHM = 60, tissue probability map = TPM.nii, voxel size = 1 × 1 × 1 mm^3^, 4th degree b-spline interpolation. FSL’s FMRIB’s Automated Segmentation Tool (FAST) was used for tissue segmentation, with the following parameters: 3 classes, segmentation smoothness = 0.1, 4 main-loop iterations, bias field smoothing extent = 20.

After tissue segmentation, the grey matter maps were spatially smoothed using SPM’s smooth function using a three-dimensional FWHM (8 mm). Grey matter maps were smoothed to minimize individual variability in sulci and gyri positioning. In other words, a pattern of regional atrophy may be undetected if there is considerable variation in sulcal anatomy in the region. Spatial smoothing is a common strategy in voxel-based morphometry for this reason as well as to render datasets that are normally distributed for subsequent analysis. Nonetheless, since CNN filters can be quite sensitive to contours, the impact of atrophy on sulcal shape may also be an important feature. For these reasons, all deep learning analyses described below were performed with smoothed as well as unsmoothed grey matter maps for comparison.

### MRI dataset group imbalance correction

In order to avoid the potential imbalance caused by a larger sample size of HC participants than patients with TLE, which could lead to classification bias or overfitting to the majority group, the synthetic minority over-sampling technique (SMOTE) was used.^17^ This approach corrects the group imbalance condition by increasing the number of minority group samples to equal the number of majority group samples. In our study, the TLE group was the minority group. Thus, we balanced the cohorts by using a *k*-nearest neighbour version of SMOTE that generates synthetic minority image samples with similar GM tissue patterns. In general, for each minority image sample $X_{m}$, SMOTE applies a two-step approach to create synthetic minority samples. First, a small subset of k image samples $\{X_{i}{\}}_{i=1}^{k}\;$in the remaining data set are identified if have similar spatial and pixel value patterns using the Euclidean distance measurement $\underset{\forall \in X\;}{\min}\sqrt{\sum (X-X_{m}{)}^{2}}\;$. Next, a sample $\hat{X}$ from $\{X_{i}{\}}_{i=1}^{k}\;$is randomly selected and a synthetic minority image sample $X_{s}=X_{m}+\left(X_{m}-\hat{X}\right)*R_{d}$ is estimated where $R_{d}$ is a displacement value that is randomly selected from uniform distribution with mean equal to zero and standard deviation equal to one. These two steps are repeated until the number of minority samples equals the number of majority samples. The results reported in Section 3 use five nearest neighbours (i.e. *k* = 5).

It should be emphasized that the imbalance correction is a key step during the approach for out-of-sample predictions. Considering the sample used in this study (MTLE *n* = 95 and controls *n* = 203): without imbalance correction, the training group would have approximately 68% of controls. As such, a training model could ‘learn’ this imbalance and achieve 68% accuracy by simply predicting all individuals on the testing group as controls. With imbalance correction, the number of controls and patients in the training group is the same and the imbalance is not taken into account to predict the test group. This important step in the approach also underscores the importance of evaluating predictive values for each group, in addition to accuracy alone.

### Deep-learning classification model

**Figure 1:**
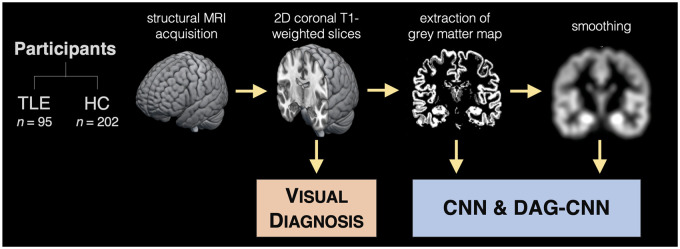
**Summary of the methodological approach.** This study evaluated 95 patients with left medial temporal lobe epilepsy (TLE) and 202 healthy controls (HC) from across three epilepsy centres. They all underwent 3 T structural MRI acquisition. The coronal slices were employed by human experts (epileptologists and neuroradiologists) to visually diagnose each scan into either group while blind to the correct label. Coronal slices were then processed to extract the grey matter tissue. Both the raw and smoothed grey matter of sequential coronal slices were fed into a convolutional neural network (CNN) and a direct acyclic graph CNN (DAG-CNN) to probe the accuracy of a machine learning approach.

The overall approach is summarized in Fig.  1 and detailed in the Materials and Methods section below. A supervised deep-learning (DL) approach to MRI data was applied to classify individuals into one of two group labels (HC or TLE). CNN and DAG-CNN classification models were used to identify GM tissue patterns in MRI data that could recognize HC individuals or individuals with TLE. Both classifications models (CNN and DAG-CNN) were created using the deep-learning MATLAB 2020a toolbox and utilized high-performance GPU computing resources to optimize computationally intensive grid-search and cross-validation analysis techniques. The details of both DL classification models are provided below. The code for these models is publicly available from https://github.com/brent-munsell/enigma_cnn (Accessed 3 December 2021).

**Figure 2:**
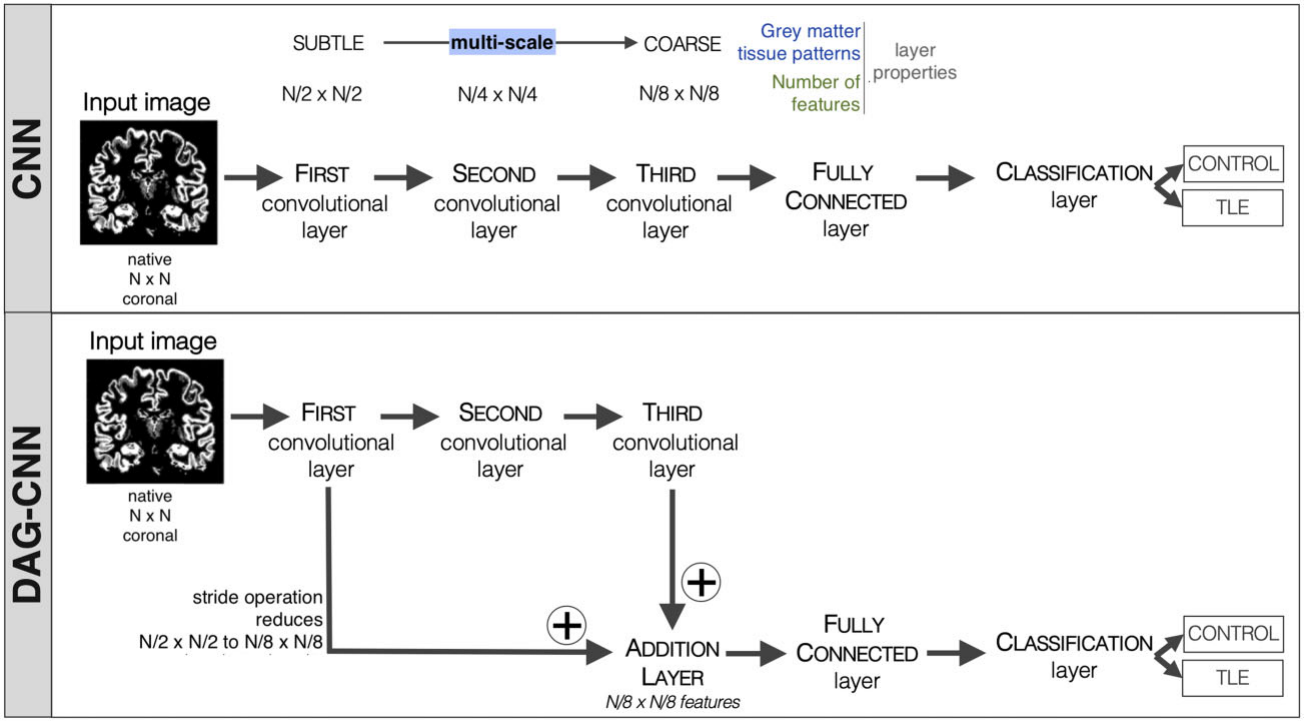
**CNN classification modelling approach.** Schematic diagram that outlines the basic operation of our CNN and DAG-CNN designs. Notice that contrary to CNN, DAG-CNN learns hierarchical grey matter tissue patterns and then combines convolution layers using an addition operation.

The CNN classification model used three convolution layers and one fully connected classification layer (Fig.  2—CNN). When an image was input into the CNN model, the layers were applied sequentially: (i) the input image was processed by the first convolution layer and the GM tissue features learned by the first convolution layer were input into the second convolution layer; (ii) subsequently, the GM tissue features learned by the second convolution layer were input into the third convolution layer; (iii) thereafter, the GM tissue features learned by the third convolution layer were input into the classification layer; and (iv) finally, the output of the classification layer was the predicted group label. The CNN classification model featured several hyperparameters that were identified by a grid-search procedure to fine-tune the model’s performance, including learning-rate, number of epochs, validation frequency, filter (or kernel) size for each convolution layer (i.e. a square n × n matrix), and number of filters. Conceptually, the CNN classification model is a pyramidal-based technique that learns GM tissue features at different scales. For instance, if a first layer applies a 2 ×2 convolution kernel to the image data the total number of GM tissue pattern features is reduced by two (assuming stride is the size of kernel with no padding). At this scale, with this kernel, features may represent subtle GM tissue patterns that may be related to precise folding patterns. However, when the second layer applies a 2 ×2 convolution kernel to the GM features found by the first convolution layer, the number of features is further reduced by two and represents more coarse GM tissue patterns that maybe localized to a specific region in the brain. Generally speaking, this multi-scale feature approach greatly simplifies the complexity of the learning approach and reduces a high-dimension problem to a lower-dimension problem.

Similarly, the DAG-CNN classification model used three convolution layers and one fully connected classification layer. However, this approach also included one addition layer (Fig.  2—DAG-CNN). Contrary to CNN, the layers in the DAG-CNN can have inputs from multiple layers, as well as outputs to multiple layers. This is evidenced by the aforementioned additional layer, i.e. two inputs, one from the first convolution layer and a second from the third convolution layer. Additionally, like the CNN model, each layer was applied, starting at the first convolution layer processing the image data and ending at the classification layer predicting the group label. Also identical to the CNN classification model, our DAG-CNN classification model featured the same hyperparameters that were identified by a grid-search procedure. Conceptually, the DAG-CNN uses a multi-scale approach to learn GM tissue patterns; however, this approach has the ability to combine subtle GM tissue features (identified in the first convolution layer) with coarse GM tissue features, and then use both feature representations for classification.

### Classification model evaluation

**Figure 3:**
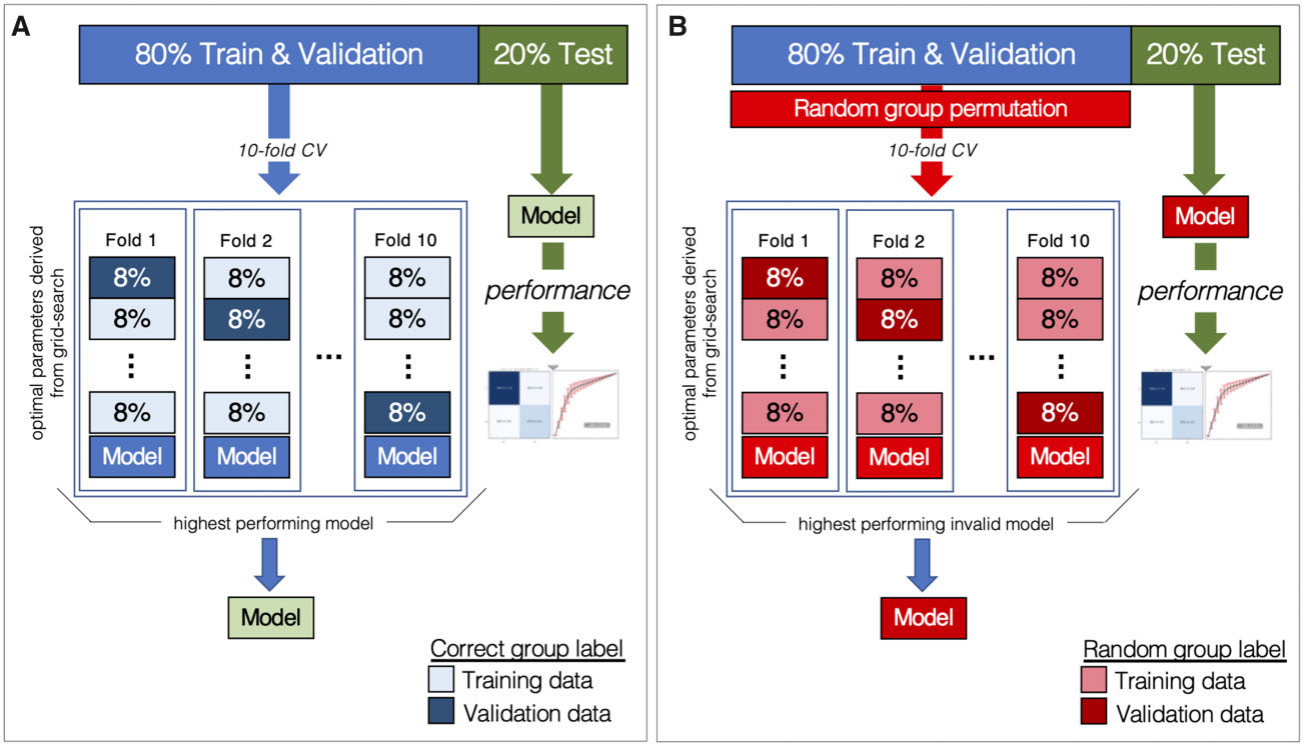
**Classification model performance evaluation.** (**A**) Diagram that illustrates our 10-fold grid search process to create a correctly trained pipeline with the highest classification accuracy, and (**B**) Diagram that illustrates our 10-fold cross-validation process to create a pipeline using shuffled labels to yield a random distribution to assess statistical significance.

Classification performance was evaluated using a 10-fold procedure (Fig.  3A) that was introduced in a recent study using machine learning to study the classification of temporal lobe epilepsy using multicentric ROI-level MRI data.^18^ More specifically, given a set $\{{X_{i}\}\;}_{i=1}^{M}$ of input images where $X_{i}$ is a 2D square resolution N × N image for participant i and M is the total number of participants, and a set $\{{l_{i}\}\;}_{i=1}^{M}$ of labels that defines the corresponding group label (e.g. HC = 0 and TLE = 1) for each participant, the following steps were sequentially applied to identify the optimal modelling parameters:

1. The image and participant label data sets were randomly shuffled together (participant image & label are maintained) and then an 80/20 percent stratified (based on participant label) split was applied to both data sets, where 80% became training and validation data and 20% becomes test data.
2. Using only the training and validation data split, a 10-fold stratified grid-search procedure was applied to our classification model. In particular, the training and validation data were equally split into ten stratified folds. Next, one-fold was selected as validation data and the remaining nine folds became the training data. Using the training & validation data, a grid-search was performed to estimate the optimal *modelling* *parameters* (i.e. deep learning network hyper-parameters) in 2D coronal plane images that yielded the highest classification accuracy. The coronal orientation was chosen because this is the most widely used plane when human experts compare side-to-side hippocampal changes to determine the presence or absence of underlying pathology. This process was repeated until each fold had been selected as the validation fold, resulting in ten classification models (i.e. one for each fold).
3. Using the ten classification models created by the grid-search procedure above, the model that had the highest classification accuracy (predicted the correct participant label the greatest number of times) was selected and its modelling parameters were identified to be optimal.
4. A classification model was constructed using the optimal modelling parameters and performance was evaluated using only the subjects in the test data. This was achieved by creating a 2 ×2 confusion matrix and then calculating the positive predictive value (PPV), negative predictive value (NPV), sensitivity (SEN), specificity (SPC), area under the curve (AUC) and accuracy (ACC).

To assess the stability of our analysis, steps 1 through 4 were repeated 1000 times. In total, 1000 confusion matrices were created that were used to compute the mean and standard deviation for each of our performance metrics (PPV, NPV, SEN, SPC, AUC and ACC).

### Assessment of statistical significance and model visualization

Statistical significance was defined by comparing the accuracies of the model with real data versus random distribution. The random distribution was obtained by shuffling the labels and repeating the training and testing process multiple times, without contamination of testing samples in the training group. Specifically, performance was evaluated using a 10-fold procedure, however, steps 2 and 3 were modified so that the training and validation participant labels were randomly permutated in step 2 and then a 10-fold stratified cross-validation procedure (no grid-search) was applied using the optimal modelling parameters, hence creating 10 random (or *invalid*) classification models (Fig.  3B). In step 4, the highest performing random classification model was selected, a random 2 × 2 confusion matrix was created, and each of our performance metrics were computed. Similarly, to assess the stability of our analysis this was repeated 1000 times. In total, 1000 random confusion matrices were created that were used to compute the mean and standard deviation for each of our performance metrics.

Lastly, statistical significance was assessed by evaluating how often the mean performance metric of the correctly trained classification models was higher than the performance metric of the random trained classification models. For instance, if the average classification accuracy of the correctly trained model was greater than 98% of the classification accuracies obtained in the random trained model, the probability that the correct model classification accuracy was merely due to chance was 2% or *P *=* *0.02. The same analysis was applied to each performance metric used in our analysis.

The 2D convolutional layers in the trained classification model were then used to *visualize* GM tissue structures that are able to differentiate TLE patients from HC subjects. In general, the output of a convolution layer represents information about neighbouring data located in a particular region in the brain. More specifically, when the convolutional layer is given input data (e.g. image data or data from a previous layer in the model) the layer creates a 2D *activation* map where values in the map indicate influence on classification performance. For instance, a large positive or negative value in an activation map suggests the input data may represent an abnormal structure in the brain that greatly influences classification performance. Since we are only concerned about the size of value, and not the sign (i.e. positive or negative), the absolute value operator is applied to the activation map. At each convolution layer in our model, N convolution operations are performed that will create N activation maps when data are input into the convolutional layer. Next, the absolute value operator was applied to the N activation maps, and the activation map with the largest summed total value (i.e. all the values in the activation map are added) was selected. Our visualization approach was applied to each classification model created by our evaluation procedure. In particular, for each image sample in the test, three activation maps were selected (one for each convolutional layer in the classification model), and then the values in the activation map were normalized to a value in [0 1] by simply identifying the largest positive value and then dividing the map by this value. This was repeated 100 times, which resulted in 1000 normalized activation layer maps at each convolutional layer. Lastly, at each layer the 1000 normalized activation layer maps were added together creating one activation map and then the values were normalized to a value in [0 1] by identifying the largest positive value and then dividing the map by this value.

### Comparison with human accuracy

A panel of epilepsy specialists (*n *=* *6 neurologists and *n *=* *1 neuroradiologist) who routinely assess and treat TLE evaluated a randomly chosen sub-sample of cases *n *=* *100, of which *n *=* *28 were TLE and *n = *72 were controls. Among the patients with left TLE in this subset, *n *=* *19 had been considered to have TLE-HS based on their clinical work-up. The panel of experts was not aware of the diagnosis of each case. They were aware that there were only cases of left TLE due to medial temporal atrophy and no other pathologies. They were also aware that there were more controls than epilepsy patients in the sample, but they did not know the percentage of each group. All experts were presented with a mosaic of evenly spaced (every 5 mm) coronal slices of the T_1_-weighted images to demonstrate the entire hippocampal formation and surrounding structures, also including more anterior and posterior planes beyond the hippocampal formation (planes −41, −36, −31, −26, −21, −16, −11, −6, −1, 4 mm, in reference to the anterior commissure). A polling system was used where each expert anonymously rated the scan as either being an HC or TLE. A majority vote was obtained based on these results. We also recorded the breakdown of individual classifications.

### TLE categories

As described above, CNN and DAG models were trained and tested with all patients with TLE grouped together to ensure that the model included features from patients with visually identified hippocampal atrophy as well as from those without such findings, since both groups may have anatomical features that could be useful for classification beyond the hippocampal region. Testing accuracies were then assessed based on all patients, but also based on specific sub-classes, namely: lesional TLE, non-lesional TLE, seizure-free TLE (gold-standard) and non-seizure free TLE. For comparison, the accuracy of classification from human interpretation was also recorded for all categories.

### Data availability

The data that support the findings of this study are available from the corresponding author, upon reasonable request.

## Results

### Patient demographics

The study included 95 with left-sided MTLE (‘TLE’) and 202 healthy controls (‘HC’). Participants were recruited from three independent epilepsy centres as described in the Materials and Methods section. As shown in Table  1, there were no significant differences between patients with TLE and controls in age and gender proportion.

**Table 1:** Summary of demographic and clinical information for patients with temporal lobe epilepsy (TLE) and control participants

	TLE	Controls	Statistical test
	*n *=* *95	*n *=* *202	
Age at surgery	39.4 (18.7)	42.3 (14.5)	*t* = 1.46, *P* = 0.14
Gender (% female)	62%	56%	χ^2^ = 4.6, *P *=* *0.31
Age at onset	16.2 (11.6)	–	
Median seizure frequency/month	5	–	
Surgery type	62% resection		
	38% laser		
Seizure freedom	60%		

Values are mean (SD) unless otherwise specified.

### Machine learning approach

The CNN classification model used three convolution layers and one fully connected classification layer (Fig.  2—CNN) while the DAG-CNN classification model also employed an addition layer (Fig.  2—DAG-CNN).

### CNN TLE versus HC classification performance

We initially performed 10-fold stratified grid-search approach to find the optimal CNN model parameters, revealing a learning rate = 0.0006, number of epochs = 160, validation frequency = once every 80 epochs, first convolution layer = 40 filters with kernel size 20 × 20, second convolutional layer = 10 filters with kernel size 10 × 10, third convolution layer = 15 filters with kernel size 20 × 20, and the optimal 2D coronal image was found in plane 113 (out of 156). The optimal model parameters were then used to generate 1000 correct 2 × 2 confusion matrices using correct CNN classification models (Fig.  3A) and 1000 incorrect 2 × 2 confusion matrices using incorrect CNN classification models (Fig.  3B). The accuracy and AUC of CNN were also significantly higher than chance: CNN accuracy = 0.85 ± 0.03 versus random model accuracy = 0.46 ± 0.12 (*P* < 0.0001); CNN AUC = 0.83 ± 0.03 versus random model AUC = 0.47 ± 0.12 (*P* < 0.0001) (Table  2).

**Table 2:** TLE versus HC classification performance and statistical significance summary based on CNN and DAG-CNN models

	Correct Classification model		Randomized Classification model		*P*-value
Metric	Mean	SD	Mean	SD	
CNN model					
Accuracy	0.85	0.023	0.46	0.0117	<0.001
Positive predictive value	0.75	0.056	0.47	0.146	0.0180
Negative predictive value	0.91	0.026	0.46	0.149	<0.001
Area under the curve	0.83	0.028	0.47	0.113	<0.001
Specificity	0.87	0.025	0.61	0.113	0.0030
Sensitivity	0.82	0.042	0.33	0.104	<0.001
DAG-CNN model					
Accuracy	0.87	0.040	0.49	0. 106	<0.001
Positive predictive value	0.84	0.070	0.55	0.257	0.2288
Negative predictive value	0.89	0.054	0.46	0.157	<0.001
Area under the curve	0.86	0.041	0.50	0.122	<0.001
Specificity	0.91	0.043	0.67	0.104	<0.001
Sensitivity	0.82	0.067	0.34	0.134	<0.001

**Figure 4:**
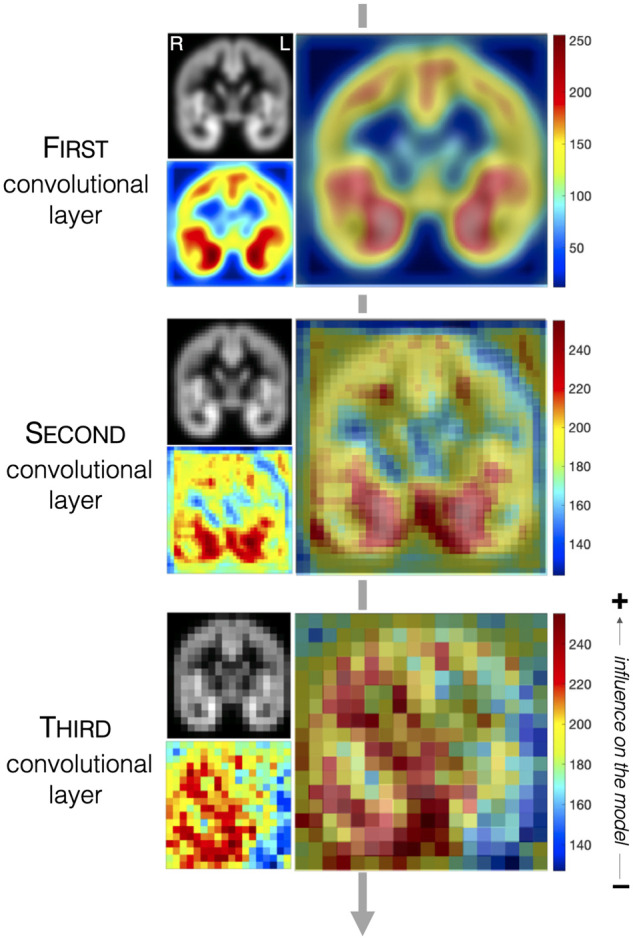
**CNN classification model visualization.** For each convolution layer, the top left figure shows the corresponding 2D coronal image, the bottom left figure shows the feature activation map (red colour represents grey matter regions that contribute most to classification accuracy), and the larger figure on the right shows the feature activation map overlaid on the 2D coronal image.

To better understand GM tissue patterns that the CNN used to differentiate TLE participants from HC, the visualization technique was applied to the three convolution layers defined in the CNN classification model (Fig.  4). In general, the grey matter volume of the motor cortex and hippocampus GM surfaces (both hemispheres) was identified by the 20 × 20 kernel in the first convolution layer; the grey matter volume of the hippocampus GM (both hemispheres) was identified by the 10 × 10 kernel in the second convolutional layer; and the grey matter volume of several right GM surfaces, which include the temporal lobe, hippocampus and motor cortex regions, was identified by the 15 × 15 kernel in the third convolutional layer. The GM surface features identified by the third convolution layer were used to differentiate TLE participants from HC and have the largest impact on classification performance (Table  2).

### DAG-CNN LTLE versus HC classification performance

The optimal DAG-CNN model parameters found by the 10-fold stratified grid-search approach were learning rate = 0.0004, number of epochs = 160, validation frequency = once every 10 epochs, first convolution layer = 10 filters with kernel size 30 × 30, second convolutional layer = 40 filters with kernel size 15 × 15, third convolution layer = 20 filters with kernel size 15 × 15, and the optimal 2D coronal image was found in plane 117 (out of 156). The optimal model parameters were then used to generate 1000 correct 2 × 2 confusion matrices using correct DAG-CNN classification models (Fig.  3A) and 1000 incorrect 2 × 2 confusion matrices using incorrect DAG-CNN classification models (Fig.  3B). The accuracy and AUC of DAG-CNN were significantly higher than chance: DAG-CNN accuracy = 0.87 ± 0.04 versus random model accuracy = 0.49 ± 0.11 (*P* < 0.0001); DAG-CNN AUC = 0.86 ± 0.04 versus random model AUC = 0.5 ± 0.12 (*P* < 0.0001) (Table  2).

**Figure 5:**
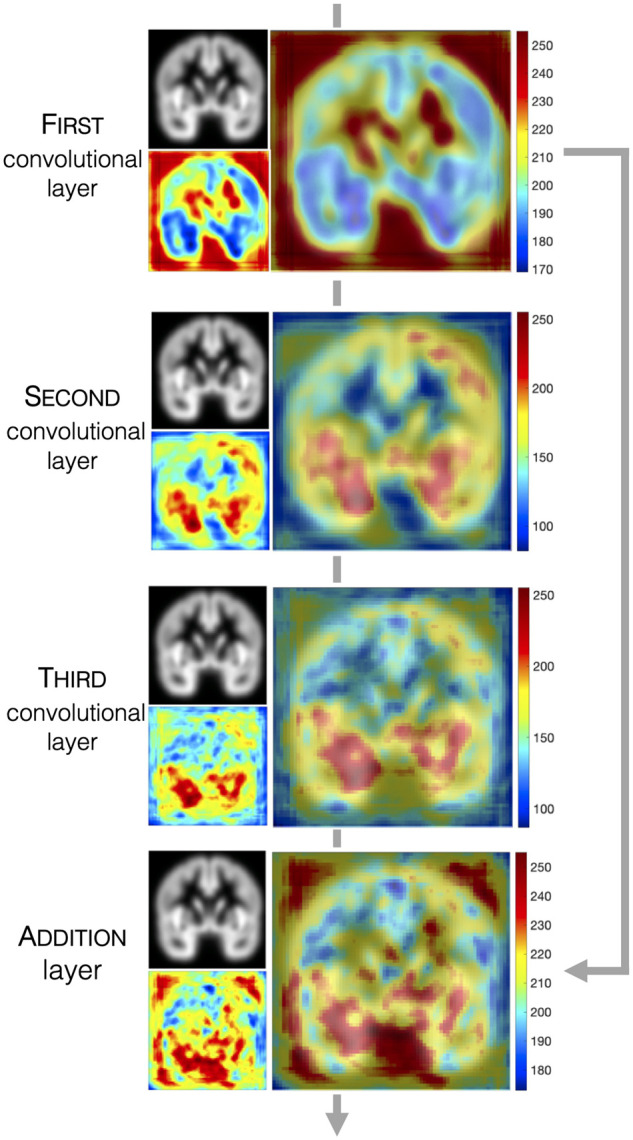
**DAG-CNN classification model visualization.** For each convolution and addition layer, the top left figure shows the corresponding 2D coronal image, the bottom left figure shows the feature activation map (red colour represents grey matter regions that contribute most to classification accuracy), and the larger figure on the right shows the feature activation map overlaid on the 2D coronal image.

To better understand GM tissue patterns that the DAG-CNN used to differentiate TLE participants from HC, the visualization technique was applied to the three convolution layers and the addition layer defined in the DAG-CNN classification model (Fig.  5). In general, the overall cortical and sub-cortical GM volumes (both hemispheres) were identified by the 30 × 30 kernel in the first convolution layer, the grey matter volume of the left motor cortex and the hippocampus (both hemispheres) was identified by the 15 × 15 kernel in the second convolutional layer, the grey matter volume of the hippocampus (both hemispheres) was identified the 15 × 15 kernel in the third convolutional layer, and then the cortical and sub-cortical grey matter (first convolution layer) combined with the hippocampus grey matter (third convolution layer) in the addition layer.

### Accuracies and predictive values per TLE category—comparison with human accuracy

**Figure 6:**
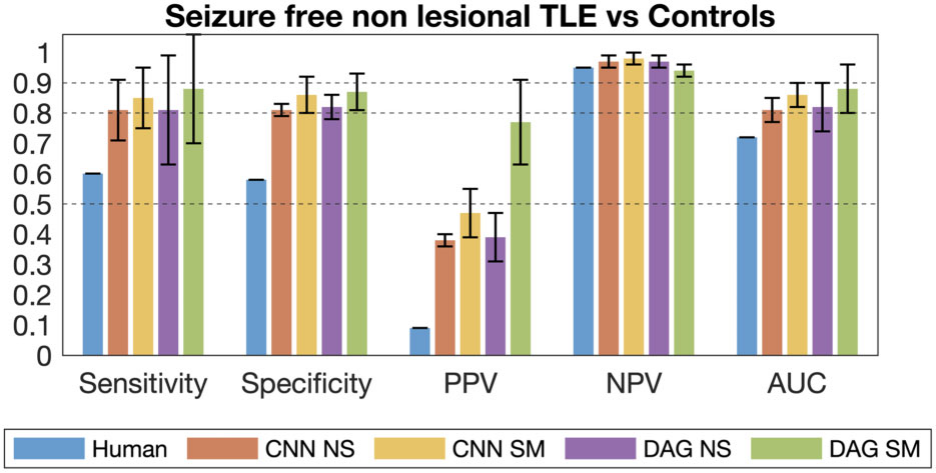
**Results for the gold standard classification.** The figure shows sensitivity, specificity, positive (PPV) and negative (NPV) predictive values, as well as the area under the curve (AUC) for the gold standard classification of HC versus non-lesional patients with TLE who had seizure freedom after surgery. Positive predictive value means the predictive value towards the identification of TLE. Negative predictive values mean the predictive value towards the identification on controls. Note the superior performance of machine learning models relative to human raters for this category. The error bars indicate 2 standard deviations. CNN NS: CNN non smoothed grey matter maps, CNN SM: CNN smoothed grey matter maps, DAG NS: DAG non smoothed grey matter maps, CNN SM: DAG smoothed grey matter maps.

The accuracies and predictive values of CNN and DAG across different categories of TLE are shown in Fig.  6 and in Supplementary Fig. 1 and Supplementary Table 1. As described in the Materials and Methods section, the categories of TLE were defined based on whether there was visually identified hippocampal atrophy and based on surgical results. Humans were able to very accurately detect patients with hippocampal atrophy, as demonstrated by the high sensitivity in lesional TLE patients versus HC. Moreover, cases predicted as controls by humans were also highly likely to be controls (high NPV). However, humans misclassified controls as patients relatively often, as demonstrated by the relatively lower specificity across all classes.

Notably, the accuracies of CNN and DAG-CNN were vastly superior to the human accuracies in cases of non-lesional TLE, including non-lesional TLE who became seizure free after surgery (Fig.  6). This last category is particularly meaningful. Seizure freedom is the gold standard, i.e. it provides the clearest marker of diagnostic confirmation of left TLE and that CNN and DAG-CNN are particularly useful in identifying these patients. The *Z*-scores of human performance in comparison with CNN and DAG performances are shown in Supplementary Table 1. Note that sensitivity among lesional cases was within 2 standard deviations of the mean of DAG and CNN. However, for non-lesional cases, the human performance was *Z* = −6.9 standard deviations below the average DAG and CNN performances for all non-lesional cases, and *Z* = −3.73 standard deviations below the average for seizure-free non lesional cases. Overall, CNN and DAG-CNN were also better at classifying controls, as demonstrated by the consistently higher specificity of all DAG and CNN models.

## Discussion

In this study, we evaluated the accuracy and the anatomically important features of neural network classifiers applied to radiological images of patients with epilepsy. Using the identification of left TLE as a foundational approach, we aimed to investigate whether CNN or DAG-CNN would be sensitive to quantifying grey matter changes in epilepsy and aid in the classification of the disease. Importantly, we used seizure freedom after surgery as the diagnostic gold-standard (i.e. confirmation of left TLE), and we were particularly interested in non-lesional cases, since these pose a particular challenge to diagnosis by human experts. Overall, we observed that CNN and DAG-CNN did not differ from human experts in terms of identifying patients with lesional TLE. However, CNN and DAG-CNN were considerably better at identifying presumed non-lesional cases. Furthermore, CNN and DAG-CNN were generally better at discriminating patients from controls (i.e. were more specific in their classification). CNN and DAG-CNN had comparable accuracies relative to each other, except that DAG-CNN had fewer false positives (i.e. patients with TLE being misclassified as controls) using smoothed grey matter maps, suggesting a higher affinity of the method for TLE-related patterns of atrophy. Relevant implications of these findings are discussed in more detail in subsequent sections.

### Classification model performance

The performance of a DAG-CNN classification model was similar to that of the CNN classification model (Table  2). Both approaches showed similar ACC, NPV, AUC, SEN and SPC performance when the model was used to predict the group label (i.e. LTLE-HS or HC) when given a coronal plane oriented 2D GM image of a participant. For all five metrics (ACC, NPV, AUC, SEN and SPC), both modelling approaches were significantly better (i.e. *P*-value < 0.05) than random models, confirming the statistical significance of our findings. The relatively high DAG-CNN PPV for smoothed images may be related to the DAG design, in particular the additional layer that combines the GM tissue features in the first and third convolution layers, likely allowing for learning of patterns otherwise imperceptible to the more linear approach of conventional CNN. That is, the overall grey matter pattern of the cerebral cortex (in the first convolution layer) may have an additive influence on classification performance than the grey matter of the hippocampus (in the third convolution layer).

From feature importance maps, both modelling approaches appeared to be identifying the same type of GM tissue patterns at the first, second, and third convolution layers (Figs.  4 and 5), and the GM tissue features that have the largest impact on classification performance localized to the hippocampus and the temporal lobe region.

### Extra-hippocampal atrophy in TLE as an important feature for classification

The classic pathological findings in TLE are cell loss, atrophy and gliosis in the hippocampus, which appear on MRI as atrophy of the hippocampal formation on T_1_-weighted images and increased T_2_-weighted hippocampal signal.^6^,^7^ While these are visually apparent in some patients, brain structural changes related to MTLE are not restricted to the hippocampus, but extend beyond the medial temporal structures and the temporal lobes.^16^,^19^ Extra-hippocampal abnormalities are not often noticeable by visual inspection, but numerous quantitative MRI studies have consistently demonstrated limbic system atrophy in the context of MTLE.^10^,^19–21^ In fact, different approaches used for brain quantification have provided converging evidence that MTLE is associated with entorhinal cortex damage, perirhinal cortex damage as well as atrophy involving the anterior cingulate, the insula, neocortical temporal and frontal structures and the thalamus, among others.^10^,^11^ In general, tissue atrophy has been found to be more pronounced in structures connected to the hippocampus. However, in spite of its prevalence in TLE, the diagnostic importance of extrahippocampal atrophy is somewhat unclear given the fact that it is seldom quantitatively defined on MRI.

Our findings indicate that many extra temporal regions exerted a high influence in terms of classification of left TLE. These findings support previous literature on the anatomical patterns of atrophy in TLE and demonstrate their importance for the diagnostic classification in the context of artificial intelligence. The pattern of atrophy beyond the hippocampus so often seen with VBM, Freesurfer, and manual morphometry studies can be harnessed for diagnosis.

### The concept of non-lesional TLE

The high accuracy of CNN in classifying presumably non-lesional TLE patients is the most important finding of this study. This observation demonstrates that human visual classification is overly reliant on hippocampal atrophy, whereas abnormalities in multiple other regions can contribute to the diagnosis of MTLE/TLE, yet remain imperceptible to our qualitative inspection. This is particularly important because these are the most challenging cases to diagnose. In fact, this finding could have the most profound implications for routine clinical practice once these promising methods are generalized and consistently validated. With our paradigm, neural networks are not proposed as a mere replacement for human judgement. On the contrary, this approach can serve as a powerful complementary decision support tool to guide subsequent investigative steps. In this vein, an important consequence of this observation is the fact that so-called ‘lesional epilepsy’ as a term may need to be revised, since it implies the finding of a visually (as in, humanly perceptible) identifiable lesion; however, computer-aided diagnosis may change this definition to include more subtle quantitative lesional patterns. This is critical for patient management, for instance, since an important aspect of determining surgical candidacy for drug-resistant epilepsy is the convergence of neurophysiological data with ‘lesional’ features on neuroimaging studies.

### Classification based on T1 atrophy

This study employed 2D image classification patterns based on spatially normalized grey matter maps. Clearly, there is a large number of other features that were not explored. For example, TLE has been associated with white matter atrophy and microstructural damage,^21^ abnormal 3D hippocampal curvature shape,^22^ cortical and subcortical thickness,^19^ T_2_ relaxation changes, etc. Based on the findings presented here, the next natural step would be the inclusion of one or more of these additional features and to test whether they further aid in the classification of TLE patients. It is also possible that multimodal imaging could provide non-redundant information and further increase classification accuracy. The high accuracy obtained from 2D images alone provides a very promising further avenue for this type of research. Importantly, as we move towards more complex image features, machine learning models will also need to demonstrate whether they are detecting a specific condition (e.g. epilepsy versus control) or associated confounders (e.g. lower intelligence quotient [IQ], long-standing mood or cognitive changes, etc.). The sensitivity and specificity of CNN in this endeavour will be achieved by (i) combining different disease populations with similar patterns of atrophy but different clinical courses (e.g. Alzheimer’s disease and temporal lobe epilepsy) while controlling for confounding variables (e.g. age), and (ii) shuffling labels to reflect an alternative clinical phenotype (e.g. age or IQ) independently of disease and comparing the accuracy of such classification when trained on disease-associated labels.

### Limitations

Besides the previously identified potential alternative approaches, there are a number of limitations to this study that must be highlighted. First, we employed a small number of human experts and future studies should expand the cohort of raters to include more numbers of specialized neuroradiologists. Importantly, the presentation of images for these experts was by means of pre-defined coronal slices that mirrored the type of input fed into the CNN models. We did not intend to probe whether machine learning outperforms human raters per se but rather show that CNN is feasible in the detection of TLE even in cases not recognized by human raters. However, future studies with larger cohorts should allow human raters to scroll in a 3D-viewer environment with the ability to zoom in/out, change windows, etc. Second, we focused on only one subset of epilepsy, i.e. left MTLE. Naturally, the approach here should be tested for right TLE, and also for other causes of epilepsy, particularly focal cortical dysplasia, which are often difficult to detect with the human eye. It should be emphasized, however, that a radiological decision support tool could be useful even if it can only be sensitive and specific for the diagnosis of TLE-related abnormalities, since TLE is prevalent and it is commonly a diagnosis that must be excluded during the work-up of challenging epilepsy cases. Third, this study did not evaluate raw native T1 images, i.e. non-processed native images. We attempted to begin from a well-defined starting point that is analogous to the approach extensively used before to detect extra-hippocampal abnormalities. Furthermore, we intended to compare images in standard space to evaluate feature importance and facilitate the comparison across subjects. Albeit simple to use, these pre-processing steps require time (for spatial normalization and tissue segmentation) and they are not routinely performed in clinical practice. Further studies should thus assess whether native raw images could achieve a similar classification performance, hence removing the need for pre-processing steps. This, in turn, would make the approach even easier to implement and distribute widely for centres across the world. Finally, as we define what best constitutes a gold-standard cohort to probe the accuracy of machine learning in the detection of TLE, we must consider the issue of changes in seizure outcome after surgery. Changes from seizure-free to non-seizure-free status have been observed in either direction up to 15% per year.^23^ We elected to choose the longest follow up time point available postoperatively as long as it was more than 12 months since surgery following the seminal Wiebe et al. controlled trial for the efficacy of epilepsy surgery^24^ but future studies could tease apart patients whose seizure outcome status did not change over time.

To summarize, convolutional image processing applied to 2D MRI images can achieve high accuracy in the identification of left TLE cases. The accuracy can be further increased by using smoothed grey matter maps and a DAG-CNN approach. Importantly, the accuracy of neural networks is considerably higher for non-lesional cases, which are notoriously difficult to diagnose based on qualitative analyses. The plots of anatomical regional feature classification importance suggest that neural networks can detect subtle patterns of atrophy both within and beyond the medial temporal region, consistent with those extensively described in the literature, and leverage these patterns for diagnosis of TLE. These are foundational findings for the ultimate goal of implementing computer-aided tools for assisting with the diagnosis of epilepsy.

## Funding

This study was supported by grants from the National Institute of Neurological Disorders and Stroke (NINDS) 1R01NS110347-01A (LB, DLD, RK) and R21 NS107739 (LB, BM, CM).

## Competing interests

The authors report no competing interests.

## Supplementary Material

fcab284_Supplementary_Data

## Abbreviations

ACC =: accuracy
AUC =: area under the curve
CNN =: convoluted neural network
DAG =: direct acyclic graph
FOV =: field of view
FWHM =: Full Width Half Maximum
GM =: grey matter
IQ =: intelligence quotient
MTLE =: medial temporal lobe epilepsy
MTS =: medial temporal sclerosis
MUSC =: Medial University of South Carolina
NPV =: negative predictive value
PPV =: positive predictive value
ROI =: region of interest
SEN =: sensitivity
SMOTE =: synthetic minority over-sampling technique
SPC =: specificity
TE =: echo time
TI =: inversion time
TLE =: temporal lobe epilepsy
TR =: repetition time
