# Supplementary material for: Radiological identification of temporal lobe epilepsy using artificial intelligence: a feasibility study
Source: Brain Commun. 2021 Dec 8;4(2):fcab284. doi: 10.1093/braincomms/fcab284 (PMC8887904; doi:10.1093/braincomms/fcab284)
Supplement: fcab284_Supplementary_Data [file fcab284_Supplementary_Data.docx]

Supplementary Materials for

**Radiological identification of temporal lobe epilepsy using artificial intelligence**

Ezequiel Gleichgerrcht*, Brent Munsell, Simon Keller, Daniel L. Drane, Jens H. Jensen, Vittoria Spampinato, Nigel P. Pedersen, Berndt Weber, Ruben Kuzniecky, Carrie McDonald, Leonardo Bonilha

*Corresponding author. Email: [gleichge@musc.edu](mailto:gleichge@musc.edu)

**This PDF file includes:**

Figs. S1

Table S1

Figure S1. Accuracies, sensitivities, specificities, predictive values, and AUCs for the different models for the different categories of TLE patients. CNN NS – CNN non smoothed gray matter maps, CNN SM – CNN smoothed gray matter maps, DAG NS – DAG non smoothed gray matter maps, CNN SM – DAG smoothed gray matter maps. Positive predictive value means the predictive value towards the identification of TLE. Negative predictive values means the predictive value towards the identification on controls. The error bars indicate 2 standard deviations.

**
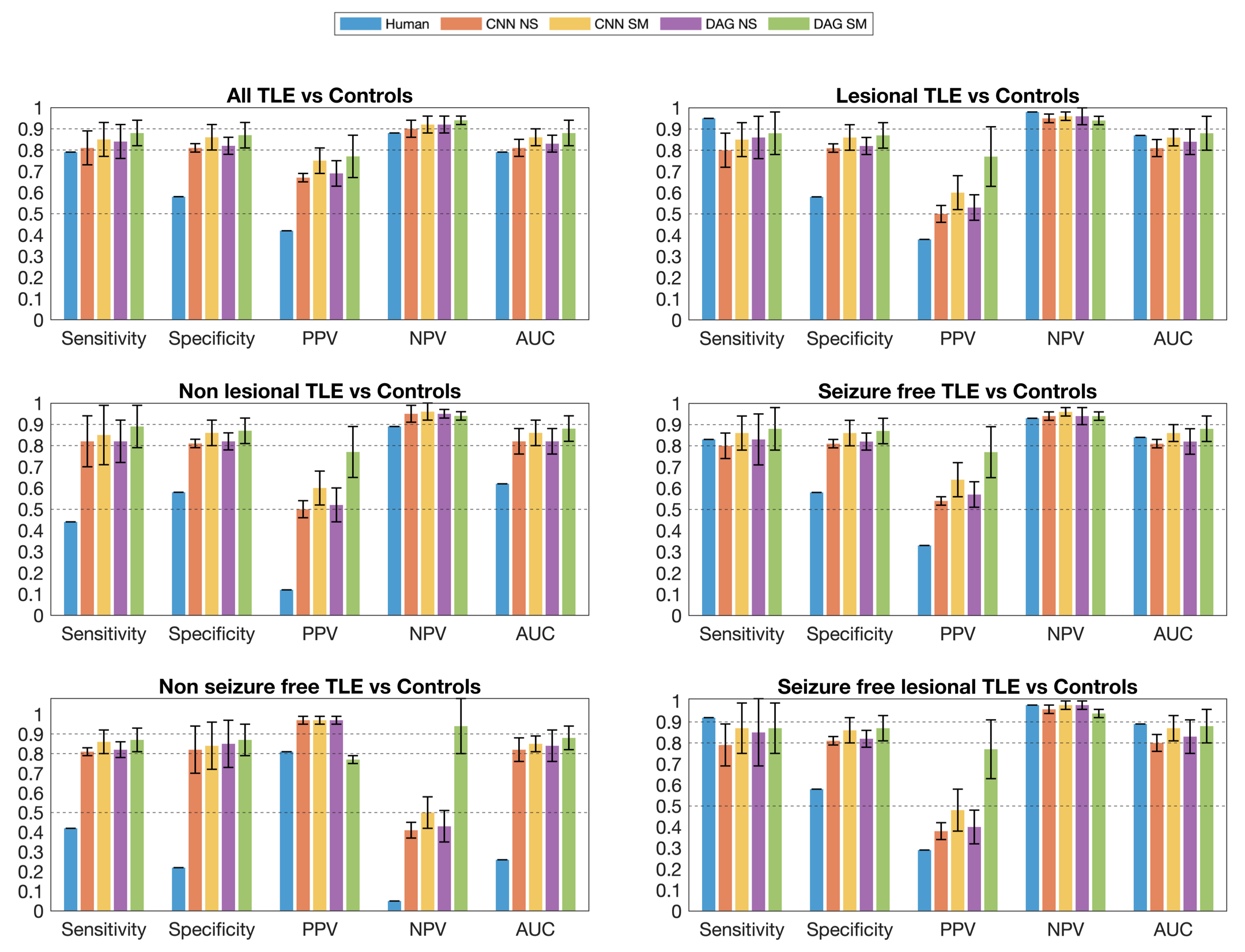
**

Table S1. Summary of human vs. artificial intelligence performance. Accuracies, sensitivities, specificities, positive predictive values (towards identifying patients), negative predictive values, and AUCs for the different models (CNN NS – CNN non smoothed gray matter maps, CNN SM – CNN smoothed gray matter maps, DAG NS – DAG non smoothed gray matter maps, CNN SM – DAG smoothed gray matter maps) for the different categories of TLE patients. Z scores displayed for human values represent the average from the Z scores in comparison with all other CNN and DAG-CNN models.

|  | | Sensitivity | Specificity | PPV | NPV | AUC |
| --- | --- | --- | --- | --- | --- | --- |
| All TLE vs Controls | | | | | | |
| Humans | | 0.79 | 0.58 | 0.42 | 0.88 | 0.79 |
|  | Z scores | -1.52 | -13.42 | -11.99 | -2.53 | -2.47 |
| CNN NS | Mean | 0.81 | 0.81 | 0.67 | 0.90 | 0.81 |
|  | Stdev | 0.04 | 0.01 | 0.01 | 0.02 | 0.02 |
| CNN SM | Mean | 0.85 | 0.86 | 0.75 | 0.92 | 0.86 |
|  | Stdev | 0.04 | 0.03 | 0.03 | 0.02 | 0.02 |
| DAG NS | Mean | 0.84 | 0.82 | 0.69 | 0.92 | 0.83 |
|  | Stdev | 0.04 | 0.02 | 0.03 | 0.02 | 0.02 |
| DAG SM | Mean | 0.88 | 0.87 | 0.77 | 0.94 | 0.88 |
|  | Stdev | 0.03 | 0.03 | 0.05 | 0.01 | 0.03 |
| Lesional TLE vs Controls | | | | | | |
| Humans | | 0.95 | 0.58 | 0.38 | 0.98 | 0.87 |
|  | Z scores | 2.20 | -13.42 | -5.44 | 2.17 | 1.11 |
| CNN NS | Mean | 0.80 | 0.81 | 0.50 | 0.95 | 0.81 |
|  | Stdev | 0.04 | 0.01 | 0.02 | 0.01 | 0.02 |
| CNN SM | Mean | 0.85 | 0.86 | 0.60 | 0.96 | 0.86 |
|  | Stdev | 0.04 | 0.03 | 0.04 | 0.01 | 0.02 |
| DAG NS | Mean | 0.86 | 0.82 | 0.53 | 0.96 | 0.84 |
|  | Stdev | 0.05 | 0.02 | 0.03 | 0.02 | 0.03 |
| DAG SM | Mean | 0.88 | 0.87 | 0.77 | 0.94 | 0.88 |
|  | Stdev | 0.05 | 0.03 | 0.07 | 0.01 | 0.04 |
| Non-lesional TLE vs Controls | | | | | | |
| Humans | | 0.44 | 0.58 | 0.12 | 0.89 | 0.62 |
|  | Z scores | -6.94 | -13.42 | -13.67 | -4.12 | -7.36 |
| CNN NS | Mean | 0.82 | 0.81 | 0.50 | 0.95 | 0.82 |
|  | Stdev | 0.06 | 0.01 | 0.02 | 0.02 | 0.03 |
| CNN SM | Mean | 0.85 | 0.86 | 0.60 | 0.96 | 0.86 |
|  | Stdev | 0.07 | 0.03 | 0.04 | 0.02 | 0.03 |
| DAG NS | Mean | 0.82 | 0.82 | 0.52 | 0.95 | 0.82 |
|  | Stdev | 0.05 | 0.02 | 0.04 | 0.01 | 0.03 |
| DAG SM | Mean | 0.89 | 0.87 | 0.77 | 0.94 | 0.88 |
|  | Stdev | 0.05 | 0.03 | 0.06 | 0.01 | 0.03 |
| Seizure free TLE vs Controls | | | | | | |
| Humans | | 0.83 | 0.58 | 0.33 | 0.93 | 0.84 |
|  | Z scores | -0.17 | -13.42 | -9.32 | -1.05 | 0.16 |
| CNN NS | Mean | 0.80 | 0.81 | 0.54 | 0.94 | 0.81 |
|  | Stdev | 0.03 | 0.01 | 0.01 | 0.01 | 0.01 |
| CNN SM | Mean | 0.86 | 0.86 | 0.64 | 0.96 | 0.86 |
|  | Stdev | 0.04 | 0.03 | 0.04 | 0.01 | 0.02 |
| DAG NS | Mean | 0.83 | 0.82 | 0.57 | 0.94 | 0.82 |
|  | Stdev | 0.06 | 0.02 | 0.03 | 0.02 | 0.03 |
| DAG SM | Mean | 0.88 | 0.87 | 0.77 | 0.94 | 0.88 |
|  | Stdev | 0.05 | 0.03 | 0.06 | 0.01 | 0.03 |
| Non-seizure free vs Controls | | | | | | |
| Humans | | 0.42 | 0.22 | 0.81 | 0.05 | 0.26 |
|  | Z scores | -21.95 | -11.24 | -10.86 | -12.90 | -19.83 |
| CNN NS | Mean | 0.81 | 0.82 | 0.97 | 0.41 | 0.82 |
|  | Stdev | 0.01 | 0.06 | 0.01 | 0.02 | 0.03 |
| CNN SM | Mean | 0.86 | 0.84 | 0.97 | 0.50 | 0.85 |
|  | Stdev | 0.03 | 0.06 | 0.01 | 0.04 | 0.02 |
| DAG NS | Mean | 0.82 | 0.85 | 0.97 | 0.43 | 0.84 |
|  | Stdev | 0.02 | 0.06 | 0.01 | 0.04 | 0.04 |
| DAG SM | Mean | 0.87 | 0.87 | 0.77 | 0.94 | 0.88 |
|  | Stdev | 0.03 | 0.04 | 0.01 | 0.07 | 0.03 |
| Seizure free lesional vs Control | | | | | | |
| Humans | | 0.92 | 0.58 | 0.29 | 0.98 | 0.89 |
|  | Z scores | 1.36 | -13.42 | -4.42 | 1.63 | 1.60 |
| CNN NS | Mean | 0.79 | 0.81 | 0.38 | 0.96 | 0.80 |
|  | Stdev | 0.05 | 0.01 | 0.02 | 0.01 | 0.02 |
| CNN SM | Mean | 0.87 | 0.86 | 0.48 | 0.98 | 0.87 |
|  | Stdev | 0.06 | 0.03 | 0.05 | 0.01 | 0.03 |
| DAG NS | Mean | 0.85 | 0.82 | 0.40 | 0.98 | 0.83 |
|  | Stdev | 0.08 | 0.02 | 0.04 | 0.01 | 0.04 |
| DAG SM | Mean | 0.87 | 0.87 | 0.77 | 0.94 | 0.88 |
|  | Stdev | 0.06 | 0.03 | 0.07 | 0.01 | 0.04 |
| Seizure free non-lesional vs Control | | | | | | |
| Humans | | 0.60 | 0.58 | 0.09 | 0.95 | 0.72 |
|  | Z scores | -3.73 | -13.42 | -12.79 | -1.76 | -4.17 |
| CNN NS | Mean | 0.81 | 0.81 | 0.38 | 0.97 | 0.81 |
|  | Stdev | 0.05 | 0.01 | 0.01 | 0.01 | 0.02 |
| CNN SM | Mean | 0.85 | 0.86 | 0.47 | 0.98 | 0.86 |
|  | Stdev | 0.05 | 0.03 | 0.04 | 0.01 | 0.02 |
| DAG NS | Mean | 0.81 | 0.82 | 0.39 | 0.97 | 0.82 |
|  | Stdev | 0.09 | 0.02 | 0.04 | 0.01 | 0.04 |
| DAG SM | Mean | 0.88 | 0.87 | 0.77 | 0.94 | 0.88 |
|  | Stdev | 0.09 | 0.03 | 0.07 | 0.01 | 0.04 |
